# Trend Analysis of Head and Neck Neoplasms between 2012–2018 in Patients Residing in Al-Madinah, Saudi Arabia: A Retrospective Study

**DOI:** 10.1055/s-0040-1722090

**Published:** 2021-02-23

**Authors:** Maha T. Alsharif, Alla T. Alsharif, Majid A. Krsoum, Mazen A. Aljohani, Osama M. Qadiri, Abdulkarim A. Alharbi, Sadeq A. Al-Maweri, Saman Warnakulasuriya, Saba Kassim

**Affiliations:** 1 Oral Diagnostic Sciences Department, Faculty of Dentistry, King Abdulaziz University, Jeddah, Saudi Arabia; 2 Preventive Dental Sciences, Taibah University Dental College and Hospital, Al-Madinah Al-Munawwarah, Saudi Arabia; 3 Taibah University Dental College and Hospital, Al-Madinah Al-Munawwarah, Saudi Arabia; 4 Prince Mohammed bin Abdulaziz Hospital, Ministry of National Guard - Health Affairs, Al-Madinah Al-Munawwarah, Saudi Arabia; 5 Ministry of Health, Salah Al-Din Primary Health Center, Ha’il, Saudi Arabia; 6 Department of Oral Medicine and Diagnostic Sciences, AlFarabi Colleges of Dentistry and Nursing, Riyadh, Saudi Arabia; 7 Department of Oral Medicine, Oral Pathology and Oral Radiology, Faculty of Dentistry, Sana’a University, Sana’a, Yemen; 8 Faculty of Dentistry, Oral & Craniofacial Sciences, King’s College London, London, United Kingdom; 9 World Health Organization Collaborating Centre for Oral Cancer, London, United Kingdom

**Keywords:** head and neck neoplasms, trend analysis, diagnostic delays, histopathology, Saudi Arabia

## Abstract

**Objectives** This study sought to present a view of head and neck neoplasms (HNN) prevalence with specific focus on sociodemographic determinants in Al-Madinah Province, Saudi Arabia.

**Materials and Methods** This was a hospital-based retrospective study based on retrieval of histopathological data for a period of 6 years between 2012 and 2018. Data was collected from the archives of the Oral and Maxillofacial Pathology Laboratory at King Fahad Hospital (the only referral center for biopsy services) in Al-Madinah City.

**Statistical Analysis** An independent *t* -test and/or nonparametric (Mann–Whitney U test, chi-squared test) tests were used to determine the differences between groups. Statistical significance was set at the *p* -value < 0.05.

**Results** Out of 96 patients, a total of 58 patients had valid biopsy data with preoperative diagnosis. Over three quarters of the cases ( *n* = 44) were benign with only 24% were malignant. Males were more likely to be diagnosed with a benign tumor than females (54.5 vs. 45.6%, respectively), and malignancy was also more common in males (64.3 vs. 24.1). A significant difference was found in relation to mean age of older patients who were more likely to be diagnosed with malignant tumors ( *p* = 0.001).

**Conclusions** The findings suggest that most of biopsied HNN cases are benign neoplasms. Age is a significant risk factor for head and neck malignancy in this region. Delays in diagnosis of HNN need to be explored.

## Introduction

Head and neck neoplasms (HNN) include a heterogeneous group of tumors, both benign and malignant predominantly affecting the upper aerodigestive track. 1 In the United States, head and neck cancer accounts for 3% of all cancers. 2 Based on the data collated and compiled at the International Agency for Research on Cancer, anatomical sites including the lip, tongue, and mouth with 355,000 newly diagnosed cases per year could be considered to carry a high proportion of head and neck cancers. 3 Among the head and neck cancers (HNC), the epidemiology of oral cancer has almost exclusively been studied in Saudi population where risk factors vary widely in different regions. 4 6 However, very little is known about the magnitude of the risk factors, morbidity and mortality of HNN (benign and malignant) in Saudi Arabia. 6 In the context of different geographic locations, knowledge about the HNN distribution and its variability within certain community profile (i.e., gender and age) is an essential prerequisite. The current literature elucidates noticeable regional differences in patterns of incidence of HNN in Saudi Arabia. 7 10 This geographic variation could be attributed to cultural differences and associated risk factors. 11 Availability of such data can help in prioritization and provision of population-specific healthcare services, implementation of preventive strategies, and distribution of resources according to specific population needs. 12

Saudi Arabia is one of the largest Arab countries of the Arabian Peninsula that is divided into 6 regions and 13 provinces. 13 The highest incidence rate of HNC (oral cancer) was reported in the southern province of Jazan, Saudi Arabia. In Jazan, oral squamous cell carcinoma is currently the most common malignancy diagnosed in females and second-most common in males. 14 Use of smokeless tobacco (shamma) has been a recognized risk factor for the high incidence of oral cancer in women in this region. 14 In another study, by Al-Zahrani et al, investigating outpatient palliative care at a major tertiary hospital in Saudi Arabia, HNC was the second most common cancer among patients (15.3%). 5 However, in the northern region, benign conditions were diagnosed more frequently than malignancies. 9 Al-Madinah Province is located in the western region of Saudi Arabia, with a population of 2,132,679. 13 Since 2009, King Fahad Hospital in Al-Madinah City is the only specialized referral center that offers free oral and maxillofacial consultative and diagnostic services throughout Al-Madinah Province and other peripheral hospitals in the region. The hospital offers aforementioned healthcare services to all patients that minimizes access bias contributing to greater accuracy on the incidences of neoplasms in this region. Due to the lack of information regarding the prevalence of HNN in Al-Madinah Province, the present study aimed to present data on HNN prevalence with specific focus on sociodemographic determinant in Al-Madinah Province, Saudi Arabia.

## Materials and Methods

### Study Design and Subjects

This was a retrospective study based on the primary data retrieved from the archives of the Oral and Maxillofacial Pathology Laboratory at King Fahad Hospital in Al-Madinah City.

All cases with confirmed histopathology analysis of a HNN (ICD-10: C00-C44, D00-D23) over a period of 6 years (2012–2018) were included in the study. Patients who were diagnosed with jaw cysts, inflammatory lesions, reactive lesions, or those with no histopathological diagnosis were excluded.

### Data Collection

The data collection sheet was designed by Microsoft Office Excel 2016 to enter information from King Fahad Hospital’s archives. All data were collected and checked by two investigators. Electronic search was generated first, then manual extraction of the data was done to make sure no data were missed. A temporary user platform was created by the hospital’s IT department with restricted access to identifiable health information for protection of patients' privacy. Other information included the following: demographic data (sex, age and nationality), date of admission to the hospital with length of stay, preoperative diagnosis, histological diagnosis, and biopsy site. Data related to presence of multiple lesions of a patient was recorded based on the history of recurrent lesions or two or more lesions on different sites of the same patient. All of the biopsies were performed by oral and maxillofacial surgeons and submitted for histopathological examination by certified general pathologists at King Fahad Hospital.

Data collection sheet involved two parts: the first part was a collection of demographic data and preoperative diagnoses, including all patients with lesions, tumors, neoplasms, cancer, and some metastasized conditions. The second part was a collection of final histopathological diagnoses according to the biopsies undertaken. Both incisional and excisional biopsies were included, and if the same patient had an incisional and excisional biopsy, the excisional biopsy was counted. Sociodemographic differences between malignant (C00-C44) and benign (D00-D23) neoplasms were assessed. The anatomic sites were further categorized into the four most common locations; the other locations were grouped under “others.”

### Statistical Analysis

The data were imported from Excel Sheet into the Statistical Package for Social Sciences (SPSS Inc., Chicago, Illinois, United States) version 20 for analysis. Descriptive analysis was performed to report the demographic variables of the patients. Means and standard deviations were reported for the continuous variables with normal distribution, and as median and Interquartile when no adherence to normality was observed (Kolmogorov–Smirnov, *p* < 0.05). Frequencies and percentages [F(%)] were used for categorical variables. An independent *t* -test and/or nonparametric (Mann–Whitney U test, chi-squared test) tests were used to determine the differences between groups. Statistical significance was set at the *p* -value < 0.05.

### Ethical Consideration and Confidentiality

The protocol for the study was approved by Taibah University’s Dental College Ethical Research Committee (IRB approval no. TUCDREC/20170920/Alsharif) and the Ministry of Health (approval no. IRB-116), Al-Madinah, Saudi Arabia. Due to the retrospective nature of the study, patients' informed consent was not required. However, confidentiality of the information was assured, that is, every patient was assigned a code.

## Results

### Patients’ Characteristics

Out of 96 patients, a total of 58 patients had valid biopsy data with preoperative diagnosis and final histopathology diagnosis of a HNN. A total of 58 HNN cases met the inclusion criteria. Table 1 shows the characteristics of the subjects: the majority (70%) were of Saudi nationality with a slight male predominance (56.9%). The mean age of the patients was ~39 ± 19 years old. Multiple head and neck lesions were found in four (6.9%) of the subjects. On average, the patients remained in the hospital for 3 days after undergoing a biopsy. Table 1 also shows that 9 patients (15.5%) were readmitted to King Fahad hospital after being discharged.

**Table 1:** Characteristics of patients with head and neck neoplastic lesions (n = 58)

Characteristics		Frequency (%)
Gender	Males	33 (56.9)
	Females	25 (43.1)
Nationality	Saudi	40 (69)
	Non-Saudi	18 (31)
Multiple sites	Yes	4 (6.9)
	No	54 (93.1)
Readmission	Yes	9 (15.5)
	No	49 (84.5)
Length of stay (d) Median (IQR)		3 (4.3)
Abbreviation: IQR, interquartile range.		

Table 2 also illustrates that males were more likely to be diagnosed with a benign tumor than females (54.5 vs. 45.6%, respectively), and malignancy was also more common in males (64.3 vs. 24.1%). The mean time between the first examination and final diagnosis for all lesions is found in Table 2 . The average time frame of “door to diagnosis” for patients diagnosed with benign lesions was ~42.3 ± 67.7 days and for malignant 29.9 ± 30.8 days.

**Table 2:** Distribution of head and neck neoplasm based on lesions (n = 58)

Head and neck lesions	*n* (%)	Gender, *n* (%)		Age range/years
		Male	Female	
**Benign neoplasms**				
Pleomorphic adenoma	7 (12.1)	3 (42.9)	4 (57.1)	24–59
Ossifying fibroma	7 (12.1)	4 (57.1)	3 (42.9)	6–36
Ameloblastoma	5 (8.6)	4 (80)	1 (20)	28–60
Warthin tumor	4 (6.9)	2 (50)	2 (50)	26–81
Odontogenic myxoma	4 (6.9)	3 (75)	1 (25)	14–26
Acquired melanotic nevus	2 (3.4)	2 (100)	0 (0)	35–63
Central giant cell lesion	2 (3.4)	1 (50)	1 (50)	17–35
Squamous papilloma	2 (3.4)	2 (100)	0 (0)	27
Lipoma	2 (3.4)	1 (50)	1 (50)	41–53
Cementoma	1 (1.7)	0 (0)	1 (100)	57
Solitary fibrous tumor	1 (1.7)	0 (0)	1 (100)	64
Pilomatricoma	1 (1.7)	0 (0)	1 (100)	5
Lobular capillary hemangioma	1 (1.7)	0 (0)	1 (100)	40
Cavernous hemangioma	1 (1.7)	0 (0)	1 (100)	10
Unspecified benign neoplasm	4 (6.9)	2 (50)	2 (50)	25–54
Total (%)/mean ± SD	44 (75.9)	24 (54.5)	20 (45.6)	5–81
Malignant neoplasms				
Squamous cell carcinoma	5 (8.5)	2 (40)	3 (60)	36–66
Mucoepidermoid carcinoma	3 (5.2)	3 (100)	0 (0)	17–62
Metastatic carcinoma	2 (3.4)	1 (50)	1 (50)	79–81
Osteosarcoma	1 (1.7)	0 (0)	1 (100)	28
Malignant neoplasm of maxillary sinus	1 (1.7)	1 (100)	0 (0)	61
Non-Hodgkin lymphoma	1 (1.7)	1 (100)	0 (0)	69
Verrucous carcinoma	1 (1.7)	1 (100)	0 (0)	57
Total (%)/mean ± SD	14 (24.1)	9 (64.3)	5 (35.7)	17–81
Abbreviation: SD, standard deviation.				

### Tumors’ Sites and Trend Over Time

**Fig. 1:**
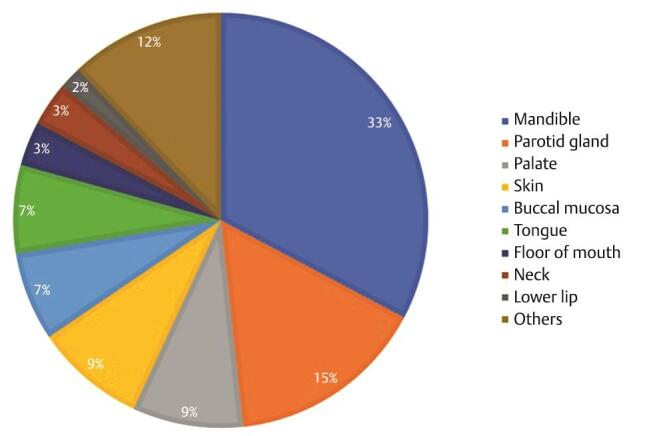
Distribution of head and neck tumors based on anatomic location.

As shown in Fig. 1 , the most frequently affected anatomical site was the mandible ( *n* = 19, 32.8%), with 94.7% of benign lesions followed by the parotid gland ( *n* = 9, 15.5%), palate and facial skin ( *n* = 5, 8.6%).

**Fig. 2:**
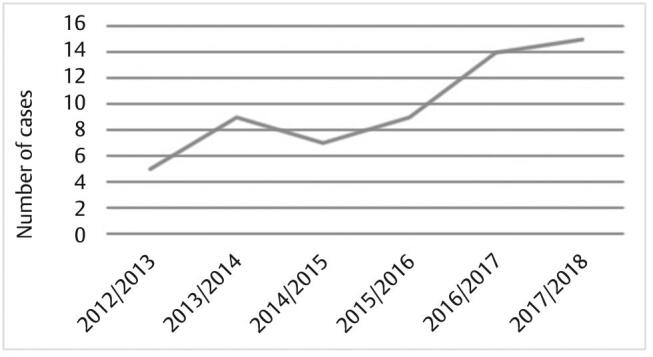
Incidence for head and neck neoplasms over the period 2012 to 2018.

Trend analysis of the data commencing in 2012 showed an increased incidence of head and neck neoplastic lesions in 2013/14 ( Fig. 2 ), with subsequent gradual decline in prevalence. A sharp increase in the number of new cases was observed from 7 cases to 15 cases per year from 2016 to 2018 but regarded statistically nonsignificant ( *p* > 0.05).

### Bivariate Analyses of Factors in Relationship to Malignant and Benign Lesions

In bivariate analyses, some of the variables revealed statistically significant differences ( *p* < 0.05) in relation to lesion diagnosis ( Table 3 ). A significant difference was found in relation to mean age of older patients who were more likely to be diagnosed with malignant tumors ( *p* = 0.001). The hospital stay of patients with malignant lesions was extended beyond that of those with benign lesions ( *p* = 0.016).

**Table 3:** Characteristics and bivariate analysis results of comparisons of patients with benign (n = 44) and malignant lesions (n = 14)

Patients’ characteristics		Diagnosis: n (%)		*p* -Value
		Benign lesion	Malignant lesion	
Gender	Males	24 (72.7)	9 (27.3)	0.52
	Females	20 (80)	5 (20)	
Nationality	Saudi	32 (80)	8 (20)	0.27
	Non-Saudi	12 (66.7)	6 (33.3)	
Multiple sites	Yes	9 (100)	0 (0)	0.06
	No	35 (71.4)	14 (28.6)	
Readmission	Yes	7 (77.8)	2 (22.2)	0.88
	No	37 (75.9)	12 (24.1)	
Site	Mandible	18 (94.7)	1 (5.3)	0.058
	Parotid gland	6 (66.7)	3 (33.3)	
	Palate	3 (60)	2 (40)	
	Skin	5 (100)	0 (0)	
	Others	12 (60)	8 (40)	
		Diagnosis: mean ± SD		***p*** -Value
		Benign lesion	Malignant lesion	
Age (y)		33.9 ± 17.4	55.3 ± 18.5	0.001
Length of stay (d)		4.4 ±5.9	5.4 ± 2.9	0.016
Time from door-to-diagnosis (d)		42.3 ± 67.7	29.9 ± 30.8	0.610
Abbreviation: SD, standard deviation.				

## Discussion

This study examined the trends in the prevalence of HNN using the data from a tertiary care facility for the period 2012 to 2018. Besides, a predictive analysis was conducted on this dataset to determine the impact of sociodemographic factors and under reporting of HNN. To the best of our knowledge, this was the first study that assessed the trend of HNN in Al-Madinah Province and prevalence of HNN has not previously been reported. Most of the prevalence studies were mainly conducted in the Southern or Eastern provinces and were either limited to oral cancer 6 or to the study of clinical cases. 15 There is also paucity of data that describe both benign and malignant neoplasms of the head and neck in descriptive studies in the world literature. 16

Overall, the results of the present study showed that the majority of diagnosed HNN cases were benign neoplasms (particularly, pleomorphic adenoma and ossifying fibroma), while only 14 cases were malignant (mainly, squamous cell carcinoma). These findings are in line with previous studies conducted in other regions of Saudi Arabia. 8 9 17 However, this finding is different from that reported in Jazan, Saudi Arabia, where 38% of cases were malignant. 7 The high incidence of malignant cancers in the latter study can be attributed to the high prevalence of smokeless tobacco use (a well-known risk factor for oral cancer) in South-western part of Saudi Arabia. 18 19 However, the prevalence of cigarette smoking in Al-Madinah was 21.3% among adolescent males 20 and 9.8% among college-aged females. 21 In Saudi, there has been rapid growth in current electronic cigarette use over the past few years, particularly among young adults. 22 This rapid increase in tobacco use would have a significant impact on the future burden of disease in this region. E-cigarettes pose serious health hazards, including increased risk for heart and respiratory disease but its causal effects on human cancer would not be known for a few decades. Themes commonly used in antismoking messages may be effective in educating the public about the potential harm of e-cigarettes. 23

Additionally, the mandible was the most frequently affected site for benign lesions, followed by the parotid gland. Intercountry comparisons revealed contradicting results citing lip and buccal mucosa as common sites for benign lesions. 4 Pleomorphic adenoma was one the most common tumors found among benign neoplasms. 24 Among salivary gland neoplasms similar findings were reported by Tian et al. 25 The etiology of salivary gland tumors is unknown and they comprise 3% of head and neck tumors in the United States. 26

A significant difference was found in relation to mean age of patients with the older people who were more likely to be diagnosed with malignant tumors. This finding consolidates the findings of previous studies. 18 27 Age is a known risk factor for head and neck malignancies. 28 Like many developing nations, Al-Madinah population age-structure is most of younger cohorts. 10 This composition has a significant impact on the present pattern/type of HNN and possible future trends. With modernization, this demographic transition is certainly underway, and the total number of people will increase as will the proportion of older people. Subsequently, the cancer burden that mainly affect the elderly will also grow.

The mean interval from door-to-diagnosis for malignant lesions was estimated close to 30 days. This delay is considered unacceptable and efforts should be taken to explore the reasons for diagnostic delay and to bridge the gaps in getting the diagnostic service to report any malignancy within 2 weeks. There is a need for primary care studies on oral cancer diagnosis. The paucity of primary care research in this area in the global literature was recently highlighted by Grafton-Clarke. 29

As stated above, this is one of the few studies that documented HNN neoplasms in Saudi Arabia. This study emphasizes the importance of oral health promotion and disease prevention programs that is specifically designed to improve oral health literacy and the quality of life of the population in this region. However, the study has some limitations worth highlighting. This was a retrospective, hospital-based study and some essential data were lacking (e.g., different modifiable and nonmodifiable risk factors that contribute to HNN such as socioeconomic status, tobacco, oral hygiene, nutritional factors, inherited syndromes, and immunological conditions). 30 31 Importantly, the nationality of the patients would be underpinned with further investigation of ethnicity/origin as this might influence the HNN. This is based on that many Saudi nationals are of different ethnicity/origin backgrounds. Also, the number of the studied sample is considered relatively low as only clinically suspected tumors are referred for histopathological examination. Alternatively, limited awareness or late biopsy seeking behavior for HNN or the paucity of specialties in the required fields may be the reason for this low histopathologic reporting. Furthermore, referred patients may have sought care elsewhere as the maxillofacial pathology service is just recently established at King Fahad Hospital in Al-Madinah. In addition, the representativeness of the study findings to whole population of the Al-Madinah is questionable as lifestyle risk factors and treatment seeking behaviors may differ by the regions. Out of 96 patients, a total of 58 patients had valid biopsy data with preoperative diagnosis. Therefore, there is a need to biopsy all suspected neoplastic conditions, as recommended in other Saudi Arabian studies. 7

## Conclusion

When a primary care physician or a dentist is confronted with a head and neck tumor, the adage “common things happen commonly” should be applied. We report here the frequency of head and neck tumors found in a pathology service that would be useful for improving referral services in this region of Saudi Arabia. Diagnostic delays encountered demand strengthening of guidelines for referral of suspected cancers in the community.
